# Feeding Experience Affects the Behavioral Response of Polyphagous Gypsy Moth Caterpillars to Herbivore-induced Poplar Volatiles

**DOI:** 10.1007/s10886-016-0698-7

**Published:** 2016-05-12

**Authors:** Andrea C. McCormick, Andreas Reinecke, Jonathan Gershenzon, Sybille B. Unsicker

**Affiliations:** Department of Biochemistry, Max Planck Institute for Chemical Ecology, Hans-Knöll-Straße 8, 07745 Jena, Germany; Institute of Agriculture and Environment, Massey University, Private Bag 11222, 4442 Palmerston North, New Zealand; Department of Evolutionary Neuroethology, Max Planck Institute for Chemical Ecology, Hans-Knöll-Straße 8, 07745 Jena, Germany; Department of Behavioral Ecology and Evolutionary Genetics, Max Planck Institute for Ornithology, Eberhard-Gwinner-Str. 7, 82319 Seewiesen, Germany

**Keywords:** Herbivore-induced plant volatiles (HIPV), Behavioral plasticity, Host-plant selection, Polyphagous insects, Lepidoptera, Salicaceae

## Abstract

Plant volatiles influence host selection of herbivorous insects. Since volatiles often vary in space and time, herbivores (especially polyphagous ones) may be able to use these compounds as cues to track variation in host plant quality based on their innate abilities and previous experience. We investigated the behavioral response of naïve (fed on artificial diet) and experienced (fed on poplar) gypsy moth (*Lymantria dispar*) caterpillars, a polyphagous species, towards constitutive and herbivore-induced black poplar (*Populus nigra*) volatiles at different stages of herbivore attack. In Y-tube olfactometer assays, both naïve and experienced caterpillars were attracted to constitutive volatiles and volatiles released after short-term herbivory (up to 6 hr). Naïve caterpillars also were attracted to volatiles released after longer-term herbivory (24–30 hr), but experienced caterpillars preferred the odor of undamaged foliage. A multivariate statistical analysis comparing the volatile emission of undamaged plants vs. plants after short and longer-term herbivory, suggested various compounds as being responsible for distinguishing between the odors of these plants. Ten compounds were selected for individual testing of caterpillar behavioral responses in a four-arm olfactometer. Naïve caterpillars spent more time in arms containing (*Z*)-3-hexenol and (*Z*)-3-hexenyl acetate than in solvent permeated arms, while avoiding benzyl cyanide and salicyl aldehyde. Experienced caterpillars avoided benzyl cyanide and preferred (*Z*)-3-hexenyl acetate and the homoterpene (*E*)-4,8-dimethyl-1,3,7-nonatriene (DMNT) over solvent. Only responses to DMNT were significantly different when comparing experienced and naïve caterpillars. The results show that gypsy moth caterpillars display an innate behavioral response towards constitutive and herbivore-induced plant volatiles, but also that larval behavior is plastic and can be modulated by previous feeding experience.

**Electronic supplementary material:**

The online version of this article (doi:10.1007/s10886-016-0698-7) contains supplementary material, which is available to authorized users.

## Introduction

Plant-derived cues play a role in host-plant selection by herbivorous insects and influence behaviors crucial to insect fitness and survival, such as feeding, mating, and oviposition (Bruce et al. 2005; Carrasco et al. 2015). In 1978, Jaenicke hypothesized that host-choice in phytophagous insects is mainly the responsibility of ovipositing females, who should choose plants that would maximize survival and performance of their offspring (“preference-performance” or “mother knows best” hypothesis) (Jaenike 1978). However, numerous studies in the past have demonstrated that this is not always the case (Barbosa et al. 1989; Hunter 1995; Roff 1990). Furthermore a recent meta-analysis revealed a strong positive correlation between oviposition preference and larval performance in oligophagous species, while this was not the case for polyphagous species (Gripenberg et al. 2010). These findings suggest that the larvae of polyphagous insects must be under high selective pressure to make their own host choices.

For polyphagous larvae feeding on deciduous trees, it might be especially important to have host-selection ability, since deciduous plants are subject to seasonal changes in which the quality and availability of resources at oviposition time vary greatly from those at hatching time and throughout the larval lifespan. Among other traits, variations in plant quality involve alterations in nutrient content, tissue toughness, and the amounts of plant defense chemicals. Variation in plant availability may affect competition for shelter and resources as well as predation pressure (Dixon 1970, 1975; Dixon et al. 1993; Feeny 1970; Singer et al. 2004). All these factors might force larvae to move from one feeding site to another several times during the life cycle.

In this complex and changing environment, it is advantageous for polyphagous larvae to recognize cues that indicate alterations of quality, distribution, and availability of plant resources. Healthy plants constantly release a number of volatiles in relatively low amounts that confer their characteristic smell (constitutive compounds). However, the amount and composition of these volatile blends is dramatically altered in response to environmental changes and upon biotic and abiotic stress (Arimura et al. 2005, 2009; Dudareva et al. 2004; Pichersky et al. 2006), thus allowing insects to extract potentially valuable information on host quality.

Numerous studies have demonstrated that herbivore induced plant volatiles (HIPVs) are used by herbivore enemies to obtain information about the location and quality of their prey (Dicke and Baldwin 2010; McCormick et al. 2012; Mumm and Dicke 2010). Often, HIPVs are avoided by gravid herbivore females, which prefer to oviposit on undamaged plants, and these entail lower risks of competition for their offspring and lower amounts of herbivore-induced plant defenses (De Moraes et al. 2001; Huang et al. 2009; Kessler and Baldwin 2001; Rostás and Hilker 2002). However, HIPVs also can be used as cues by foraging caterpillars (Carroll et al. 2008; Huang et al. 2009; Landolt et al. 2000), and recent reports suggest that larvae are able to infer information about plant quality and the presence of heterospecific competitors as well as the approximate density of conspecifics based on the quantity and quality of herbivore-induced volatiles (Robert et al. 2012a, b).

The release of HIPVs is a dynamic process in which emission patterns of specific groups change during the course of herbivory giving different informational value for both herbivores and their natural enemies (Hoballah and Turlings 2005; Kugimiya et al. 2010; von Mérey et al. 2013; McCormick et al. 2014a). Green leaf volatiles (GLVs) are widespread C_6_ compounds emitted rapidly upon mechanical damage to membranes as happens during herbivore feeding. As soon as feeding stops, GLV emission also decreases making them reliable indicators of herbivore presence. In contrast, most terpenoids increase only several hours after damage in a light dependent manner, and they continue being released long after herbivores have been removed (Clavijo McCormick et al. 2014a). For this reason, they have been associated with the induction of plant defense mechanisms (Arimura et al. 2009; Gershenzon and Dudareva 2007; Mumm and Dicke 2010). Compounds derived from other biosynthetic pathways have been less studied in this regard, but in the case of poplar trees minor nitrogen containing compounds that are relevant for indirect tree defense have emission patterns closely resembling those of GLVs, whereas major nitrogenous compounds that play a role in direct defense have delayed emission patterns like terpenoids (Irmisch et al. 2013; McCormick et al. 2014a). Phenylpropanoids and other amino acid derivatives have more complex and varied emission patterns in response to herbivory, and their ecological roles in poplar trees are not yet fully understood (McCormick et al. 2014a, b).

Although plant volatiles (both constitutive and herbivore induced) are optimal cues for foraging herbivores, polyphagous larvae face the challenge of finding common volatiles that can be used as indicators of host quality over a broad range of taxonomically unrelated plants, since the complexity of the signals indicating host-plant quality for individual plant species may exceed the processing capacity of the insect (Bernays 2001). In this context, polyphagous insects may benefit from experience-dependent behavioral plasticity in their host-selection process (Anderson and Anton 2014; Bernays 2001; Carrasco et al. 2015).

The aim of this study was to investigate the effect of experience on the behavioral responses of polyphagous *Lymantria dispar* caterpillars to both constitutive and herbivore-induced volatiles emitted by black poplar (*Populus nigra*), and the potential role of individual volatile compounds as cues for foraging larvae. In order to fulfill this aim, we studied: **1**) the odor-based preferences of naïve (reared on artificial diet), and experienced (reared on black poplar leaves) early *L. dispar* instars towards volatile blends emitted by conspecific larvae and by *P. nigra* at different time points following herbivory by conspecifics; **2**) the differences in volatile emission profiles among those odor sources that were significantly discriminated by the larvae; and **3**) the behavioral responses of naïve and experienced larvae towards 10 individual herbivore-induced black poplar volatiles.

Our hypothesis is that early *L. dispar* instars rely on the odor of conspecifics when they are naïve. Being polyphagous they possibly are attracted to plant volatiles that are emitted by a wide range of potential hosts such as GLVs, and likely are repelled by compounds associated with toxic non-volatile plant defenses. However, after feeding experience, larvae ought to avoid volatile blends that indicate the presence of induced defenses, high levels of competition for food, and other potential threats such as increased predation.

## Materials and Methods

### Insects and Plants

Gypsy moth caterpillars (*Lymantria dispar*) hatched from egg masses (provided by Hannah Nadel, Animal and Plant Health Inspection Service (APHIS) of the U.S. Department of Agriculture) originating from a laboratory culture started with individuals from flightless female populations collected in North America. This culture has been maintained by periodic addition of more individuals collected from the wild, and carefully designed mating protocols to avoid the deleterious effects of inbreeding depression.

Immediately after hatching, caterpillars were divided into two groups. A “naïve” group was reared on artificial wheat germ gypsy moth diet (MP Biomedicals LLC, Illkirch, France), and an “experienced” group on black poplar leaves derived from poplar saplings (see growth conditions below). To keep leaves fresh during the experiment, small branches were freshly cut and submerged in 15 ml polypropylene Flacon tubes (VWR International, Darmstadt, Germany) filled with water and sealed with Parafilm (Bemis NA, Neenah, WI, USA) to avoid water loss. The poplar twigs and diet were replaced twice a week, and both groups were allowed to feed *ad libitum.* All caterpillars were maintained in a climate chamber at 20 °C, 60 % rel. humidity, and 16/8 hr photoperiod until used in the experiments as 2nd instars.

Black poplar (*Populus nigra*) trees were grown from stem cuttings taken from branches of 30–60-year-old trees growing in a natural floodplain forest along the Oder River in northeastern Germany (52°34′1″N, 14°38′3″E). They were raised under summer conditions (24 °C, 60 % rel. humidity, and 16 hr/8 hr light cycle) in the greenhouse in a 1:1 mixture of sand and Klasmann clay potting substrate (Klasmann-Deilmann GmbH, Germany) and regularly fertilized. When trees were used in experiments, they were approximately 1 m tall.

### Odor Sources for Choice Assays

For choice assays, 112 black poplar saplings were divided into two groups. The first half was left undamaged to be used as controls, and the second group was subjected to herbivore feeding damage. In the herbivory group, 12 sec-instars were placed on top of the leaves and secured with clip cages (1 larva per leaf). The herbivory treatment was further divided into three groups: 1) *Short-term herbivory*: *L. dispar* caterpillars were allowed to feed for up to 6 hr on leaves, 2) *Longer-term herbivory*: caterpillars were allowed to feed for 24–30 hr on leaves, 3) *After herbivore removal*: 12 sec instars were allowed to feed for 24–30 hr, and then removed from the leaves for at least 24 hr, with tests carried out between 24 and 30 hr after removing the herbivores. Saplings were kept in the climate chamber under the conditions described above until used for behavioral tests.

To test the behavior of naïve and experienced 2nd instars towards conspecific odor and frass, we allowed 12 caterpillars to feed on poplar leaves for 24 hr. After this period, we collected them along with the frass produced and placed them on top of a 15 cm petri dish covered with moistened filter paper to avoid desiccation of the frass. This treatment was compared to *clean air* (same setup as above without frass and caterpillars).

### Y-tube Olfactometer Tests

The olfactometer consisted of a 1.5 cm inner diam glass Y-tube (main segment 20 cm long, each of the two arms 14 cm), which was located inside a white box that was homogeneously illuminated with fluorescent light (Osram, Germany) (Fig. S1A). The odor sources (listed below) were placed in two 3L Dry seal® glass containers (Duran group, Germany) connected via Teflon tubing to each arm of the olfactometer. Charcoal-purified air was pumped at a rate of 0.8 L min^−1^ into each arm of the olfactometer. Flow rates through the desiccator-Y tube system were controlled with flowmeters (Key Instruments, Trevose, PA, USA) to assure equal air streams entering each arm (0.4 L min^−1^ each).

Prior to experiments, 12 fully developed leaves were cut from control trees and the petioles were placed into a 50 ml beaker with water. In the herbivory treatments, 12 leaves with feeding larvae were used in the same way. For the treatments, 1) *short-term herbivory* and 2) *longer-term herbivory*, the larvae were kept on the leaves they were feeding on (clip cages removed), but in treatment 3) larvae were removed 24–30 hr before the experiment. All assays without exception were carried out within a 6 hr time interval between 10:00 a.m. and 4:00 p.m. For treatment 1), at least 2 hr feeding was allowed before starting olfactometer tests. Due to space limitations, we were unable to use whole saplings for the behavioral essays, thus, cut leaves might have slightly different emission profiles than those of whole saplings used for the volatile collections. However, we assume these differences to be negligible and not affecting the overall outcome of the tests or validity of results.

Odor pairs were offered to the larvae as follows: 1) clean air vs. headspace of *undamaged leaves*; 2) headspace of *undamaged leaves* vs. headspace of leaves subject to *short-term herbivory*; 3) headspace of *undamaged leaves* vs. headspace of leaves subject to *longer-term herbivory;* and 4) headspace of *undamaged leaves* vs. headspace of damaged leaves 24 hr *after herbivore removal*. An additional trial included clean air vs. the headspace of *caterpillars and their frass*.

Second instars were starved overnight prior to the experiment. Preliminary experiments showed that this starvation period did not impact caterpillar survival or mobility, and that starved caterpillars are more responsive to odor cues than satiated ones.

One caterpillar at a time was placed at the opening of the Y-tube, and behavior was recorded for 5 min with a digital camera (Logitech, Germany) located above the Y-tube. Fifty caterpillars were tested with each treatment combination. A choice was recorded once the caterpillar had entered one of the two odor-permeated arms. Caterpillars that did not make a choice within five min were not included in the statistical analysis. Hence the number of replicates for each test was variable and ranged from 30 to 50. After each individual caterpillar trail, the Y-tube was rinsed with water and odorless soap and dried. The position of the odors in the olfactometer was randomized after each trial.

### Volatile Collections

We performed volatile collections for those odor comparisons in which the larvae showed a significant preference for one odor over the other, i.e., volatiles of *undamaged leaves* vs. *short-term damage,* and *undamaged leaves* vs. *longer-term damage*. For the volatile collections, 10 poplar saplings were used, 5 were left undamaged to be used as controls, and 5 were used for the herbivory treatment after placing 12 sec-instars on the plants. Two sets of 6 hr volatile collections were carried out on the same trees, a first one during the first 6 hr of herbivory, and a second from 24 to 30 hr of herbivory; the control group also was measured twice within the same time frames. Volatile collections were performed in a climate chamber under the conditions described above.

For volatile collection, the stem and leaves of each sapling were covered with a commercial polyethylene terephthalate (PET) foil bag (Toppits® Bratschlauch, Minden, Germany). Volatiles were collected using a dynamic push-pull system in which charcoal-filtered air was pumped into the PET bags at a flow rate of 1.5 L min^−1^, and a portion of the air was pumped out of the bags at a flow of 1 L min^−1^. The outgoing air passed through a trap packed with 20 mg Super Q adsorbent (ARS, Inc., Gainesville, FL, USA) to retain the volatiles. They were collected for 6 hr from 10:00 a.m. until 4:00 p.m. Volatiles were desorbed by eluting the filter with 200 μl of dichloromethane containing nonyl acetate as an internal standard (10 ng L^−1^). After each collection, leaves were photographed to estimate the amount of damage, and at the end of the second volatile collection they were cut from the saplings and weighed in order to calculate the volatile emission in relation to fresh weight for each time point.

Qualitative and quantitative volatile analysis was conducted using an Agilent 6890 Series gas chromatograph coupled to an Agilent 5973 quadrupole mass selective detector (MS, interface temp 270 °C; quadrupole temp 150 °C; source temp 230 °C; electron energy 70 eV) or to a flame ionization detector (FID) operated at 300 °C. Constituents of the volatile mixture were separated using a ZB-WAX column (Phenomenex, Aschaffenburg, Germany, 60 m × 0.25 mm × 0.15 μ film thickness) and He (MS) or H_2_ (FID) as carrier gas. A portion (1 μl) of the sample was injected in splitless mode at an initial oven temperature of 40 °C. The temperature was held for 2 min, then increased to 225 °C with a gradient of 5 °C/min, held for another 2 min, and further increased to 250 °C with 100 °C/min and a hold for 1 min. Compounds were identified by comparison of retention times and mass spectra to those of the NIST library and with authentic standards. The absolute amount of all compounds was determined based on their FID peak area in relation to the area of the internal standard using the effective carbon number (ECN) concept as described by Scanion and Willis (Scanlon and Willis 1985).

### Behavioral Experiments With Individual Volatile Compounds

To test responses of naïve caterpillars to individual host-derived compounds, 10 volatiles representing different chemical classes were selected based on the results obtained from random forest analyses (a statistical classification performed on the quantified volatile blends, see below) and compound availability. The identity, purity, source, and chemical classes of the selected compounds are summarized in Table 1. These experiments were carried out in a four-arm olfactometer since this setup enabled better quality videos by avoiding the glass reflection from the Y tubes, allowing data to be analyzed using the behavioral analysis software Ethovision XT ® (Noldus Information Technology, Wageningen, The Netherlands).

**Table 1:** Name, purity and source for the 10 volatile compounds used in this study

Compound	Purity and source	Chemical class
(*E*)-β-Ocimene Linalool	80 % Chemos 97 % Sigma-Aldrich	Terpenoid (Monoterpenes)
(*E*)-β-Caryophyllene	98.5 % Fluka	Terpenoid (Sesquiterpenes)
DMNT	98 % chemical synthesis	Terpenoid (Homoterpenes)
(*Z*)-3-Hexenyl acetate (*Z*)-3-Hexenol	98 % SAFC Global 98 % Bedoukian	Green leaf volatiles
Salicyl aldehyde	99 % Acros Organics	Aromatics
Eugenol	95 % Acros Organics	Phenylpropanoids
Benzyl cyanide 2-Methylbutyraldoxime (*E:Z, 3:1*)	95 % Acros Organics 98 % chemical synthesis (McCormick et al. 2014b)	Nitrogenous compounds

The Teflon four-arm olfactometer was located inside a white box (Fig. S1B). A digital video camera was placed on top. Incoming airflow of 0.2 L min^−1^ entered each of the four arms of the arena, and air was pumped out at the center of the arena at a flow of 0.8 L min^−1^. All selected compounds were dissolved in dichloromethane (DCM) at a concentration of 100 ng μL^−1^, and 10 μl were dispensed onto filter paper disks placed at the edges of each olfactometer arm near the air entrance points. The used doses correspond to emission rates well within the range typically emitted by the foliage of young poplar trees (10–15 g fresh weight) (as shown in Table S2). Odors were refreshed for every individual trial, and the olfactometer was washed and dried between trials.

Two opposite arms of the olfactometer contained paper discs with the selected compound dissolved in DCM, while the paper discs in the other two arms contained solvent (DCM) only as a control. Naïve and experienced caterpillars were placed individually in the center of the arena, and a video of their behavior was recorded for 5 min. We used the time spent in each arm as response parameter. For each odor, we tested a total of 30 caterpillars, but those that did not leave the center of the arena were excluded from the statistical analyses. Hence, the number of replicates varies from 21 to 25 caterpillars in the different treatments. Video data were analyzed using EthoVision XT ®.

### Statistical Analyses

Caterpillar choice data derived from Y-tube olfactometer tests were analyzed with a *Chi square test* (*χ*^2^), and the differences between the behavior of naïve and experienced caterpillars towards the offered odors were analyzed with a *Chi square test* followed by a *Phi Cramer’s V* measure of association.

To identify compounds that distinguished the pairs of volatile blends that larvae could significantly discriminate (*undamaged leaves* vs. *short-term herbivory*, *undamaged leaves* vs. *longer-term herbivory* and *short* vs. *longer-term herbivory*), we used the machine-learning algorithm ‘random forest’ (Breiman 2001) implemented in R Studio (version 0.97.551, R Studio, Boston, MA, USA). This multivariate statistical tool creates 100.000 bootstrap samples with 8 volatiles (variables) randomly selected at each node (number of variables selected is based upon the square root of all variables, *N* = 67). For each compound, the mean decrease in accuracy (MDA) is calculated together with an out of bag error (OOB) for the different experimental treatments. The MDA is an estimate of the importance of a given compound for the classification (the higher the MDA, the more important the compound is deemed), and the OOB calculation measures the prediction error of the random forest algorithm (Breiman 2001). Some advantages associated with the use of this method for volatile analysis are the inclusion of correlated data (e.g., compounds derived from common biosynthetic pathways) in the error calculation, and an equal weighting of individual compounds, reducing common biases towards highly abundant compounds (Ranganathan and Borges 2010).

To test behavioral responses of caterpillars towards individual volatiles, we compared the time spent in the DCM (control) arms vs. the time spent in the odor arms using a Wilcoxon signed-rank test for each individual compound and level of experience (naïve and experienced). A Mann–Whitney *U* test was performed to compare the time experienced and naïve caterpillars spent in the respective arms of the four-arm arena.

## Results

### Previous Experience Affects Larval Behavioral Responses Towards the Odor of Longer-Term Damaged Foliage

**Fig. 1:**
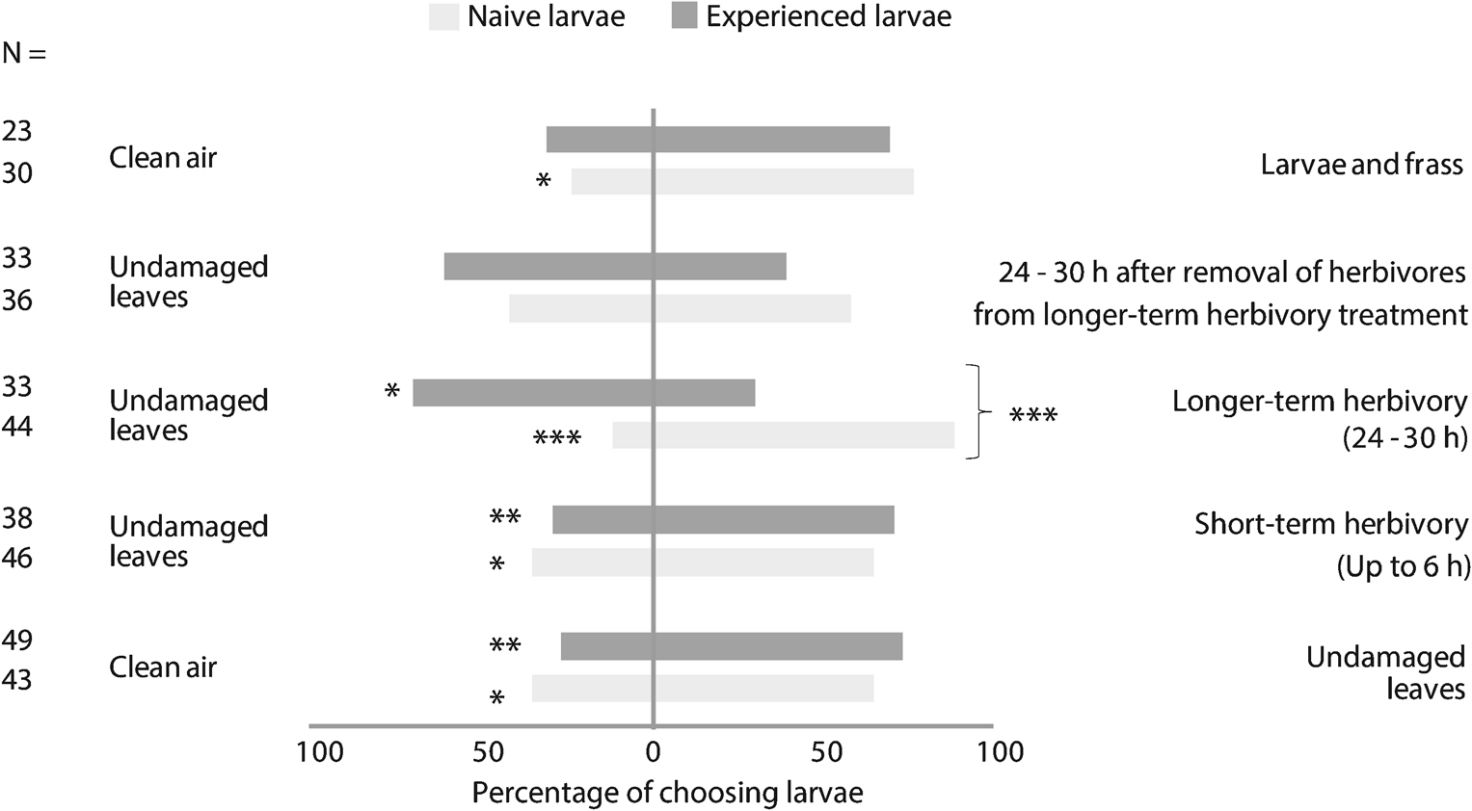
Gypsy moth caterpillar preference in Y tube olfactometer tests. Depicted is the percentage of larvae choosing each arm of a Y tube olfactometer for five different odor pairs. Light grey bars represent naïve larvae fed on artificial wheat germ diet since hatching, while dark grey bars represent experienced larvae fed on *Populus nigra* since hatching. Asterisks indicate significant differences after a *Chi square test* (*χ* ^*2*^): * = *P* < 0.05 ** = *P* < 0.01, *** = *P* < 0.001. The total number of non-choosing larvae per trial is as follows from bottom to top: *N* = 7, 1, 4, 3, 6, 6, 3, 7, 0, 7. Numbers of responding larvae per trial (*N*) ranged between 23 and 49. Further details on the statistical tests are given in Table S1

Caterpillars raised on artificial diet (“naïve”) and those that had prior experience with poplar leaves (“experienced”) both preferred the odor from *undamaged P. nigra leaves* to a *clean air* control (*N* = 43, *χ*^*2*^ = 3.93, *P* = 0.047 and *N* = 49, *χ*^*2*^ = 10.8, *P* = 0.001 for naïve and experienced larvae, respectively) (Fig. 1). They also preferred the odor from leaves after *short-term* (up to 6 hr) *herbivory* to that of *undamaged leaves* (*N* = 46, *χ*^*2*^ = 4.26, *P* = 0.039 and *N* = 38, *χ*^*2*^ = 6.74, *P* = 0.009 for naïve and experienced caterpillars, respectively). Responses of naïve and experienced caterpillars were not significantly different for either of the above comparisons (*χ*^*2*^ = 0.755, *P* = 0.385 for *undamaged P. nigra leaves* vs. *clean air* control, and *χ*^*2*^ = 0.325, *P* = 0.569 for *short-term* (up to 6 hr) *herbivory* vs. *undamaged leaves*). Further details on the statistical tests, *χ*^*2*^ and *P* values are given in Table S1.

When the odor of leaves after *longer-term herbivory* (24–30 hr) was offered against the odor of *undamaged leaves* (Fig. 1), naïve caterpillars showed a highly significant preference for the herbivore-induced black poplar odor (*N* = 44, *χ*^*2*^ = 26.27, *P* < 0.001), whereas the experienced caterpillars oriented towards the odor of *undamaged leaves* (*N* = 33, *χ*^*2*^ = 5.12, *P* = 0.024). The difference between the preferences of naïve and experienced caterpillars was significant (*N* = 77, *χ*^*2*^ = 27.73, *P* < 0.001) (Table S1). However, in tests with the odors of damaged leaves 24–30 hr *after herbivore removal* vs. *undamaged leaves*, we found no significant preference of either the naïve or experienced caterpillars towards any of these two odors. The odor of the *caterpillars and their frass* was attractive for naïve caterpillars in comparison to *clean air* (*N* = 30, *χ*^*2*^ = 8.53, *P* = 0.003), and attraction was marginally significant in the case of experienced caterpillars (*N* = 23, *χ*^*2*^ = 3.52, *P* = 0.060). No significant differences were found when comparing the preference of naïve vs. experienced caterpillars (*N* = 53, *χ*^*2*^ = 0.34, *P* = 0.561) (Fig. 1, Table S1).

### Specific Plant Volatiles are Responsible for Odor Differences Between Odor Treatments

To determine which volatiles best distinguished the headspaces of various treatments (Table S2), the machine-learning algorithm “random forest” (Breiman 2001) was employed. The importance of each compound for the distinction is expressed as the mean decrease in accuracy (MDA), and the prediction error is expressed as the out-of-bag error rate (OOB).

**Fig. 2:**
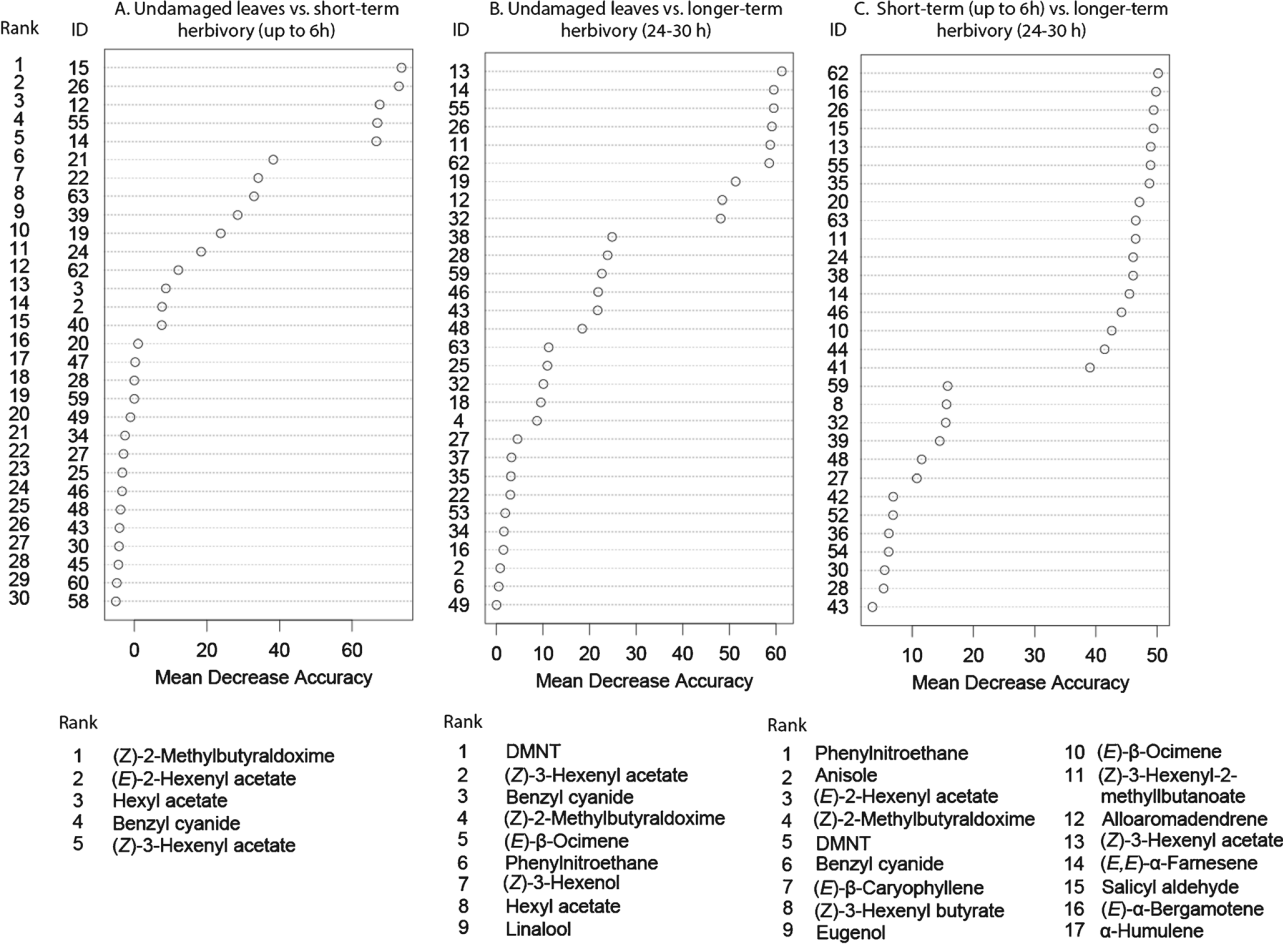
Ranking of mean decrease in accuracy (MDA) values after a random forest analysis, a statistical classification that indicates the most characteristic compounds distinguishing the volatile bouquets of the following odor pairs: **a**. *undamaged leaves* vs. leaves after *short-term* herbivory (up to 6 hr) by second instar *L. dispar* (**b**). *undamaged leaves* vs. leaves after *longer-term herbivory* (24–30 hr), and (**c**). leaves after *short-term herbivory* vs. leaves after *longer-term herbivory*. The figure shows the hierarchical rank of 30 volatile compounds for each odor pair based on their MDA. The column labeled ID indicates the identity of the volatile compounds based on their retention time as given in Table S2. The identity of the compounds having the highest MDA values for each odor pair is given at the bottom of each graph

The random forest analysis comparing the volatile blends of *undamaged leaves* and leaves after *short-term herbivory* suggested five compounds belonging to the classes green leaf volatiles and nitrogenous compounds as being responsible for the differences between these blends (Fig. 2a), with mean decrease in accuracy (MDA) values above 50 % and an out-of-bag (OOB) error rate of 0 % for each treatment. These compounds are the GLVs: (*E*)-2-hexenyl acetate, hexyl acetate, and (*Z*)-3-hexenyl acetate; and the nitrogen containing compounds: 2-methylbutyraldoxime and benzyl cyanide.

The blend of volatiles collected after *short-term herbivory* was dominated by the green leaf volatile (*Z*)-3-hexenyl acetate, which was emitted at a rate of 335.24 in ng g^−1^ FW h^−1^, three fold more than in control *undamaged leaves* (Table S2). The remaining four compounds chosen as distinctive by the random forest analysis were released in much lower amounts (at least 10 times less). All compounds present in undamaged plants were emitted in higher quantities from herbivore-damaged plants, except for 2-methylbutyraldoxime, which was not detectable in the *undamaged leaves* (Table S2).

After comparing volatile blends of *undamaged leaves* and leaves after *longer-term herbivory* (Fig. 2b), the random forest algorithm suggested nine compounds as the most important by using similar criteria as above: DMNT, (*Z*)-3-hexenyl acetate, benzyl cyanide, 2-methylbutyraldoxime, (*E*)-β-ocimene, phenylnitroethane, (*Z*)-3-hexenol, hexyl acetate, and linalool. In contrast to the comparison between *short-term herbivory* and *undamaged leaves*, the appearance of monoterpenes and a homoterpene in the list is noteworthy.

The most abundant compounds in the *longer-term herbivory* blend were (*Z*)-3-hexenyl acetate, (*E*)-β-ocimene, and DMNT with emission rates of 137.81, 101.38, and 55.29 ng g^−1^ FW h^−1^, respectively. Again, the differences between both treatments (*longer-term herbivory* and *undamaged leaves*) were mostly quantitative with higher emission in the *longer-term herbivory* treatment), except for 2-methylbutyraldoxime, which was absent from the headspace of *undamaged leaves* (Table S2).

The random forest analysis listed 17 compounds as responsible for the differences between the *short* and *longer-term herbivory* blends (Fig. 2c). Several aromatic and sesquiterpene compounds were present in the list along with others already reported above. The compounds are phenylnitroethane, anisole, (*E*)-2-hexenyl acetate, 2-methylbutyraldoxime, DMNT, benzyl cyanide, (*E*)-β-caryophyllene, (*Z*)-3-hexenyl butyrate, eugenol, (*E*)-β-ocimene, (*Z*)-3-hexenyl-2-methylbutanoate, alloaromadendrene, (*Z*)-3-hexenyl acetate, (*E,E*)-α-farnesene, salicyl aldehyde, (*E*)-α-bergamotene, and α-humulene. The *longer-term herbivory blend* had higher emission rates for most compounds except for the GLVs and a few other compounds (like the sesquiterpenes alloaromadendrene, (*E*)-α-bergamotene, and α-humulene), which were emitted in higher amounts after *short-term herbivory* (Table S2).

### Caterpillars Respond Behaviorally to Five Individual Volatiles

**Fig. 3:**
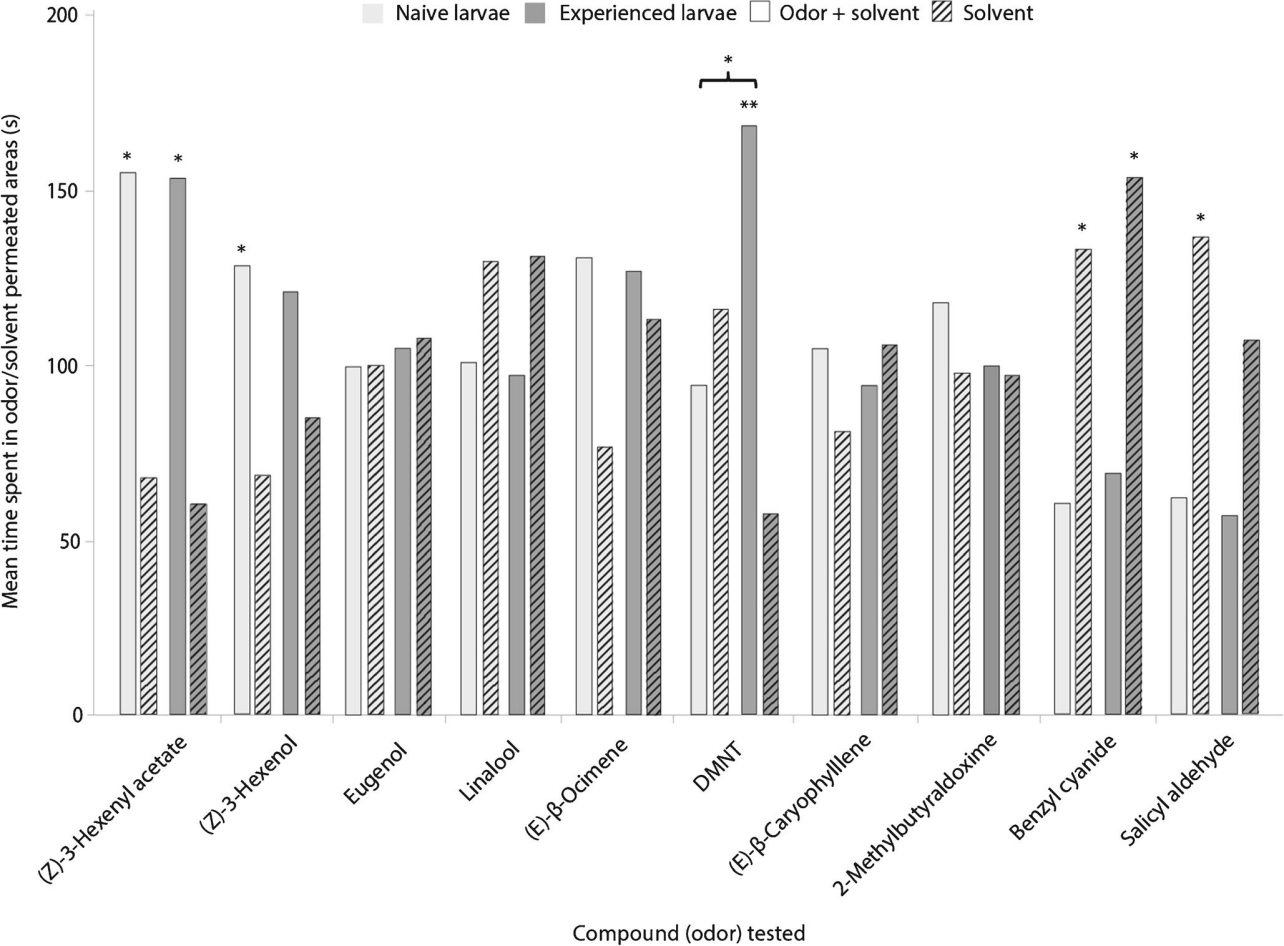
Mean time spent by naïve (*light grey bars*) and experienced (*dark grey bars*) early instar *Lymantria dispar* caterpillars in arms of a four-arm olfactometer containing two arms permeated with a solvent-control (*dichloromethane, DCM, striped bars*) and two arms permeated with a test compound (*non-striped bars*) applied to a filter paper disk at a concentration of 100 ng mL^−1^. Asterisks on top of the bars depict statistically significant differences between the time spent in the compound-containing arms and the solvent control for each compound and level of experience (naïve/experienced) tested independently (* = *P* ≤ 0.05, ** = *P* ≤ 0.01) after a non-parametric Wilcoxon test. The asterisk above the square bracket represents a significant difference between the times spent in DMNT-containing arms by naïve and experienced larvae after a non-parametric *U* Mann–Whitney test (* = *P* ≤ 0.05). Further details on the statistical tests and the comparison between the times spent in odor-permeated arms for naïve and experienced caterpillars are given in Table S3

Based on the random forest results and compound availability, 10 individual volatiles were selected for testing larval behavior. Four-arm olfactometer tests showed that caterpillars respond behaviorally to five of the 10 tested compounds (Fig. 3, Table S3). Naïve individuals spent significantly more time in arms permeated with the GLVs, (*Z*)-3-hexenol and (*Z*)-3-hexenyl acetate, than in arms with solvent controls (*N* = 24, *Z* = −2.257, *P* = 0.024 and *N* = 22, *Z* = −2.159, *P* = 0.031, respectively). In contrast, naïve caterpillars spent more time in solvent control arms than in arms permeated by either benzyl cyanide or salicyl aldehyde (*N* = 21, *Z* = −2.138, *P* = 0.033 and *N* = 24, *Z* = −2.029, *P* = 0.043, respectively).

Experienced caterpillars spent a longer time in arms permeated with either DMNT or (*Z*)-3-hexenyl acetate than in control arms (*N* = 25, *Z* = −2.623, *P* = 0.009 and *N* = 22, *Z* = −2.256, *P* = 0.024, respectively), but avoided benzyl cyanide, and they showed a strong trend towards the avoidance of salicyl aldehyde (*N* = 22, *Z* = −1.997, *P* = 0.046 and *N* = 22, *Z* = −1.834, *P* = 0.067, respectively). All other tested compounds did not significantly affect larval behavior.

When comparing the *time spent* in the odor-treated arms between naïve and experienced caterpillars, only the homoterpene DMNT showed significant differences between the two levels of experience, with experienced caterpillars spending more time in arms permeated with this compound (*N* = 49, *U* = 186, *P* = 0.022) (Table S3).

## Discussion

The results show that naïve early instars of gypsy moth are attracted by volatiles released from conspecific larvae and from undamaged and herbivore-damaged black poplar leaves. However, previous feeding experience affects these responses. Naïve larvae (fed on artificial diet) preferred volatiles from herbivore-damaged leaves to those from undamaged leaves, but experienced larvae (fed on black poplar foliage) preferred volatiles from undamaged leaves to those from leaves on which conspecifics had fed for longer time periods. Individual bioassays on 10 selected volatile compounds suggest five compounds: (*Z*)-3-hexenol, (*Z*)-3-hexenyl acetate, benzyl cyanide, salicyl aldehyde, and DMNT to be involved in the odor-based choices of the larvae.

Attraction of early instars towards herbivore-infested plants previously has been reported for other lepidopteran species, e.g., *Ostrinia furnacalis* (Huang et al. 2009), *Spodoptera frugiperda* (Carroll et al. 2008), and *Cydia pomonella* (Landolt et al. 2000). However, attraction towards HIPVs may entail risks, as herbivore-infested plants harbor competitors, and herbivory induces both direct and indirect defenses (Howe and Schaller 2008; McCormick et al. 2012; Mumm and Dicke 2010). Nevertheless, caterpillars may benefit from having an aggregation behavior during earlier instars due to feeding facilitation, improved microclimate, or reduced probability of predator attack (Reader and Hochuli 2003; Zalucki et al. 2002). In this sense, HIPVs may indicate the presence of conspecifics and act as aggregation kairomones (Loughrin et al. 1995).

Additionally, freshly hatched gypsy moth larvae may be under selection pressure to quickly find any sort of host plant, since adult females have low mobility and are not highly selective in their oviposition choices (Alison and Elkinton 2000; Barbosa 1978; Barbosa and Capinera 1978; Doane and McManus 1981; Lance and Barbosa 1981). Some adult females are even flightless, especially in North America where the insect was introduced from Europe in the middle 19th century (Keena et al. 2008). To avoid starvation, young caterpillars should not be too selective with respect to damaged and undamaged host plants. Our observation that naïve caterpillars were highly attracted to constitutive and herbivore-induced poplar volatiles, even after long-term attack from conspecifics, supports this argument.

In contrast to naïve caterpillars, those experienced on black poplar foliage avoided the blend of black poplar volatiles after *longer-term* (24–30 hr) *L. dispar* herbivory. This behavior suggests that experienced caterpillars associate herbivore-induced volatiles with negative nutritional aspects, such as increases in toxic or otherwise detrimental plant secondary metabolites or increased competition. The observation supports the hypothesis that previous experience and behavioral plasticity cause polyphagous insects to respond to changes in resource quality and availability (Bernays 2001). Other studies have reported that previous feeding experience has a significant effect on behavioral responses towards HIPVs (von Mérey et al. 2013), and that previous experience can affect decision making well into the adult stages (Blackiston et al. 2008). It also has been found that caterpillars avoid volatile cues associated with failed predation events (Low et al. 2014).

Naïve caterpillars were not only significantly attracted to plant-derived compounds, but also to the volatiles released by conspecific caterpillars and their frass. The presence of conspecifics may be a reliable food cue for freshly hatched caterpillars without previous feeding experience. However, our results indicate that experienced larvae also tended to prefer the odor of conspecifics and their frass over clean air, and that their overall choice is not significantly different from that of naïve caterpillars. This suggests that recognition of conspecifics remains important even after feeding experience. There are a number of advantages associated with aggregation including the reduction of the effect of host defenses, lower predation risk, and an improved microclimate (Reader and Hochuli 2003). Thus, it may be advantageous for a caterpillar to search for a host plant where conspecifics are present.

Other gregariously feeding lepidopteran caterpillars such as *Malacosoma* spp. and *Hemileuca oliviae* also track their conspecifics, but do so via silk trail aggregation pheromones (Capinera 1980; Fitzgerald 1976; Peterson 1988). For other tree-feeding insect herbivores e.g., willow leaf beetles *Plagiodera versicolora* (Yoneya et al. 2010), and the cottonwood leaf beetle, *Chrysomela scripta* (Kendrick and Raffa 2006), gregarious early instars use a combination of volatiles emitted by plants upon conspecific-damage and larval as well as frass odors from conspecifics as aggregation cues. However, these responses may be modified with development (Yoneya et al. 2010).

Previous work with *L. dispar* has characterized volatiles emitted from caterpillars and their frass after feeding on poplar. Some compounds were plant-derived, and others probably derived solely from the insect (McCormick et al. 2014a). However, the identity of the compounds causing caterpillar attraction has not yet been determined.

When the effect of individual poplar volatiles on gypsy moth caterpillar behavior was tested, five elicited behavioral responses. However, these results must be interpreted cautiously since the context in which volatiles are perceived by insects may affect their behavioral response. For instance, behavior towards single compounds may differ from that towards odor blends (Webster et al. 2010), and compounds that are not attractive when tested individually may become attractive in the presence of background odors (Beyaert et al. 2010). Furthermore, Insects have different sensitivities to different compounds and are capable of distinguishing between various concentrations, and they respond differently to them (Hansson and Stensmyr 2011; Martin et al. 2011; Todd and Baker 1999). Below we summarize previous knowledge about compounds that elicit behavioral responses, and speculate about what information they may convey to foraging *L. dispar* larvae.

Benzyl cyanide is a nitrogen-containing compound barely detectable in the constitutive blend of poplar. It is emitted locally at the site of herbivore damage after attack, but emission drops shortly after the herbivore is removed (McCormick et al. 2014a, b). Benzyl cyanide attracts natural enemies of *L. dispar* under field conditions (McCormick et al. 2014b), and its semi-volatile precursor phenylacetaldoxime, which accumulates in poplar leaves upon herbivory, has been demonstrated to decrease *L. dispar* survival and performance when offered at natural concentrations in an artificial diet (Irmisch et al. 2013). These observations suggest that benzyl cyanide might be a good indicator of reduced food plant quality and a higher risk of predation, and are consistent with the avoidance of this compound by both naïve and experienced gypsy moth larvae in our olfactometer studies.

The emission of the aromatic compound salicyl aldehyde also may indicate reduced host plant quality. Salicyl aldehyde emission correlates positively with the concentration of a well-known group of anti-herbivore defense compounds of the Salicaceae known as salicinoids or phenolic glycosides (Unsicker, SB, unpublished results) that negatively affect the fitness of generalist insect herbivores, while salicinoids act as feeding stimulants and oviposition cues for specialists (Boeckler et al. 2011; Keefover-Ring et al. 2014). The avoidance of salicyl aldehyde and benzyl cyanide by naïve larvae in our studies and the avoidance (or strong trend towards avoidance) of these compounds by experienced larvae support the hypotheses that larvae innately avoid compounds that indicate potentially toxic food sources, and that this ability is independent of previous experience.

Rather than avoidance, both naïve and experienced gypsy moth caterpillars were attracted to GLVs, such as (*Z*)-3-hexenyl acetate and (*Z*)-3-hexenol, which are taxonomically widespread compounds emitted by plants in copious amounts after wounding caused by herbivores or mechanical injury (Arimura et al. 2009; Mumm and Dicke 2010; ul Hassan et al. 2015). These observations suggest that polyphagous gypsy moth caterpillars use widespread volatiles as cues of potential host plants.

In addition to GLVs, terpenoids are another class of volatiles released from numerous plant species upon herbivore damage (Dudareva et al. 2004; Gershenzon and Dudareva 2007; Maffei et al. 2011). However we observed no behavioral response from naïve larvae towards individually tested terpenes. This might have several explanations: 1) the compounds are not perceived; 2) behavioral responses to the compounds occur only after they have been associated with positive or negative stimuli; or 3) responses depend on other compounds of the blend or background odors (Beyaert et al. 2010; Webster et al. 2010). The attractiveness of DMNT to experienced caterpillars in our experiments depended upon previous experience, supporting the second assumption.

A large group of terpenoids is released from black poplar foliage, so it is possible that other terpenes not tested in this study may be more relevant as host cues for polyphagous tree-feeding insects than the tested compounds. Such is the case of the hemiterpene isoprene, which was not detectable by the methods used here, but is among the most abundant volatiles released by poplar and other tree species, making it an ideal cue for tree-feeding herbivores (Laothawornkitkul et al. 2009; Schnitzler et al. 2010).

The homoterpene DMNT is one of the most abundant herbivore-induced volatiles in poplar and other plant species upon damage by a wide range of herbivores (Carroll et al. 2006; Gossner et al. 2014; McCormick et al. 2014b; Oluwafemi et al. 2013). However, its emission profile contrasts with those of most other poplar terpenoid volatiles that are first emitted only several hours after damage, and continue to be emitted for several hours and even days after the herbivore is no longer present. DMNT is first emitted just after the onset of herbivory, and emission falls to low levels within few hours after herbivores are removed (McCormick et al. 2014a). This emission pattern makes DMNT a reliable indicator of both the timing and intensity of herbivory. Low emissions may signal the presence of a suitable food source (being consumed by other herbivores), whereas high emissions might indicate increased competition with con- and heterospecifics and induced plant defenses from long-lasting herbivore damage.

Other herbivores also exploit DMNT as a host plant recognition cue, such as in cowpea seedlings where DMNT is the major volatile induced by *Spodoptera frugiperda* herbivory (Carroll et al. 2008). Caterpillars of this species are behaviorally attracted to DMNT, and this attraction increases when DMNT is supplemented with plant odors from healthy plants. Several studies have found DMNT associated with herbivore enemy attraction (e.g., Dicke et al. 1990; Tamiru et al. 2011; Turlings et al. 1995), suggesting that it also may serve as an indicator of potential predation risk.

In summary, our results support the hypothesis that naïve early instars of *L. dispar* are attracted to the odor of conspecifics and compounds that are emitted by a large number of potential host plants such as the green leaf volatiles. At the same time, they avoid compounds associated with toxic plant defenses such as benzyl cyanide and salicyl aldehyde. Previous feeding experience modifies behavioral responses to herbivore-induced volatile blends and individual volatiles, and may allow larvae to detect increased competition for food resources and higher levels of direct as well as indirect chemical plant defenses. Further research is needed to assess larval response towards blends rather than individual odors, and to determine the influence of larval development on odor preference.

## Electronic supplementary material

Below is the link to the electronic supplementary material.

Fig. S1 Detail of the setups used for behavioral assays. A. Y tube olfactometer with glass vessels, and B. Four-arm olfactometer. (DOCX 4954 kb)

Table S1 Results of Chi square test for Y-tube olfactometer essays and Pearson Chi square test for comparison of behavioral responses between naïve and experienced caterpillars followed by 2 × 2 Phi-Cramer’s V test. (DOCX 15 kb)

Table S2 Volatile emission from leaves of undamaged poplar saplings (control) and saplings fed upon by second instar Lymantria dispar larvae (LD2) up to 6 hr (Short-term herbivory), and between 24 and 30 hr (longer-term herbivory). Values are displayed in ng g-1 FW h-1. The reason why two controls are shown is because the same plants were measured at an initial stage of herbivory (up to 6 hr of damage) and between 24 and 30 hr of damage, and so were the corresponding controls (undamaged plants). Bold compounds were used for behavioral assays. (DOCX 31 kb)

Table S3 Results of a Wilcoxon signed rank test on the time spent by gypsy moth larvae in areas of an olfactometer permeated with a solvent or the tested odor and U Mann–Whitney test for comparison between naïve and experienced caterpillar behavior. Z and P values are given for comparisons between the odor and control treated arms for each level of experience (naïve or experienced) tested independently, and U and P values are given for comparisons between experienced and naïve caterpillars for each compound. (DOCX 17 kb)

## References

[CR1] Alison FH, Elkinton JS (2000). Effects of synchrony with host plant on populations of a spring-feeding lepidopteran. Ecology.

[CR2] Anderson P, Anton S (2014). Experience-based modulation of behavioural responses to plant volatiles and other sensory cues in insect herbivores. Plant Cell Environ.

[CR3] Arimura G, Kost C, Boland W (2005). Herbivore-induced, indirect plant defences. Biochim Et Biophys Acta-Mol Cell Biol Lipids.

[CR4] Arimura G, Matsui K, Takabayashi J (2009). Chemical and molecular ecology of herbivore-induced plant volatiles: Proximate factors and their ultimate functions. Plant Cell Physiol.

[CR5] Barbosa P (1978). Host plant exploitation by the gypsy moth, *Lymantria dispar*. Entomol Exp Appl.

[CR6] Barbosa P, Capinera J (1978). Population quality, dispersal and numerical change in the gypsy moth, *Lymantria dispar* (L.). Oecologia.

[CR7] Barbosa P, Krischik V, Lance D (1989). Life-history traits of forest-inhabiting flightless Lepidoptera. Am Midl Nat.

[CR8] Bernays EA (2001). Neural limitations in phytophagous insects: implications for diet breadth and evolution of host affiliation. Annu Rev Entomol.

[CR9] Beyaert I, Wäschke N, Scholz A, Varama M, Reinecke A, Hilker M (2010). Relevance of resource-indicating key volatiles and habitat odor for insect orientation. Anim Behav.

[CR10] Blackiston DJ, Silva Casey E, Weiss MR (2008). Retention of memory through metamorphosis: can a moth remember what it learned as a caterpillar?. PLoS One.

[CR11] Boeckler GA, Gershenzon J, Unsicker SB (2011). Phenolic glycosides of the Salicaceae and their role as anti-herbivore defenses. Phytochemistry.

[CR12] Breiman L (2001). Random forests. Mach Learn.

[CR13] Bruce TJA, Wadhams LJ, Woodcock CM (2005). Insect host location: a volatile situation. Trends Plant Sci.

[CR14] Capinera J (1980). A trail pheromone from silk produced by larvae of the range caterpillarHemileuca oliviae (Lepidoptera: Saturniidae) and observations on aggregation behavior. J Chem Ecol.

[CR15] Carrasco D, Larsson MC, Anderson P (2015). Insect host plant selection in complex environments. Curr Opin Insect Sci.

[CR16] Carroll MJ, Schmelz EA, Meagher RL, Teal PEA (2006). Attraction of Spodoptera frugiperda larvae to volatiles from herbivore-damaged maize seedlings. J Chem Ecol.

[CR17] Carroll MJ, Schmelz EA, Teal PEA (2008). The attraction of Spodoptera frugiperda neonates to cowpea seedlings is mediated by volatiles induced by conspecific herbivory and the elicitor inceptin. J Chem Ecol.

[CR18] De Moraes CM, Mescher MC, Tumlinson JH (2001). Caterpillar-induced nocturnal plant volatiles repel conspecific females. Nature.

[CR19] Dicke M, Baldwin IT (2010). The evolutionary context for herbivore-induced plant volatiles: beyond the ‘cry for help’. Trends Plant Sci.

[CR20] Dicke M, Van Beek TA, Posthumus MA, Ben Dom N, Van Bokhoven H, De Groot A (1990). Isolation and identification of volatile kairomone that affects acarine predatorprey interactions Involvement of host plant in its production. J Chem Ecol.

[CR21] Dixon AFG, Watson AE (1970). Quality and availability of food for a sycamore aphid population. Animal populations in relation to their food resources.

[CR22] Dixon AFG (1975). Effect of population density and food quality on autumnal reproductive activity in the sycamore aphid, *Drepanosiphum platanoides* (Schr.). J Anim Ecol.

[CR23] Dixon AFG, Wellings PW, Carter C, Nichols JFA (1993). The role of food quality and competition in shaping the seasonal cycle in the reproductive activity of the sycamore aphid. Oecologia.

[CR24] Doane CC, McManus ML (1981). The gypsy moth: research toward integrated pest management.

[CR25] Dudareva N, Picherski E, Gershenzon J (2004). Biochemistry of plant volatiles. Plant Physiol.

[CR26] Feeny P (1970). Seasonal changes in Oak leaf tannins and nutrients as a cause of spring feeding by winter moth caterpillars. Ecology.

[CR27] Fitzgerald TD (1976). Trail marking by larvae of the eastern tent caterpillar. Science.

[CR28] Gershenzon J, Dudareva N (2007). The function of terpene natural products in the natural world. Nat Chem Biol.

[CR29] Gossner MM, Weisser WW, Gershenzon J, Unsicker SB (2014). Insect attraction to herbivore-induced beech volatiles under different forest management regimes. Oecologia.

[CR30] Gripenberg S, Mayhew PJ, Parnell M, Roslin T (2010). A meta-analysis of preference-performance relationships in phytophagous insects. Ecol Lett.

[CR31] Hansson BS, Stensmyr MC (2011). Evolution of insect olfaction. Neuron.

[CR32] Hoballah ME,Turlings TCJ (2005) The role of fresh versus old leaf damage in the attraction of parasitic wasps to herbivore-induced maize volatiles. J Cheml Ecol 31:2003–2018 10.1007/s10886-005-6074-7 16132209

[CR33] Howe GA, Schaller A, Schaller A (2008). Direct defenses in plants and their induction by wounding and insect herbivores. Induced plant resistance to herbivory.

[CR34] Huang CH, Yan FM, Byers JA, Wang RJ, Xu CR (2009). Volatiles induced by the larvae of the Asian corn borer (Ostrinia furnacalis) in maize plants affect behavior of conspecific larvae and female adults. Insect Sci.

[CR35] Hunter A (1995). The ecology and evolution of reduced wings in forest macrolepidoptera. Evol Ecol.

[CR36] Irmisch S, McCormick AC, Boeckler GA, Schmidt A, Reichelt M, Schneider B, Block K, Schnitzler JP, Gershenzon J, Unsicker SB, Köllner TG (2013). Two herbivore-induced cytochrome P450 enzymes CYP79D6 and CYP79D7 catalyze the formation of volatile aldoximes involved in poplar defense. Plant Cell.

[CR37] Jaenike J (1978). On optimal oviposition behavior in phytophagous insects. Theor Popul Biol.

[CR38] Keefover-Ring K, Ahnlund M, Abreu IN, Jansson S, Moritz T, Albrectsen BR (2014). No evidence of geographical structure of Salicinoid chemotypes within *Populus tremula*. PLoS One.

[CR39] Keena MA, Côté MJ, Grinberg PS, Wallner WE (2008). World distribution of female flight and genetic variation in *Lymantria dispar* (Lepidoptera: Lymantriidae). Environ Entomol.

[CR40] Kendrick AP, Raffa KF (2006). Sources of insect and plant volatiles attractive to cottonwood leaf beetles feeding on hybrid poplar. J Chem Ecol.

[CR41] Kessler A, Baldwin IT (2001). Defensive function of herbivore-induced plant volatile emissions in nature. Science.

[CR42] Kugimiya S, Shimoda T, Tabata J, Takabayashi J (2010) Present or past herbivory: a screening of volatiles released from Brassica rapa under caterpillar attacks as attractants for the solitary parasitoid, *Cotesia vestalis*. J Chem Ecol 36:620–628 10.1007/s10886-010-9802-6 20490899

[CR43] Lance D, Barbosa P (1981). Host tree influences on the dispersal of first instar gypsy moths, *Lymantria dispar* (L.). Ecol Entomol.

[CR44] Landolt PJ, Brumley JA, Smithhisler CL, Biddick LL, Hofstetter RW (2000). Apple fruit infested with codling moth are more attractive to neonate codling moth larvae and possess increased amounts of (E, E)-alpha-farnesene. J Chem Ecol.

[CR45] Laothawornkitkul J, Taylor JE, Paul ND, Hewitt CN (2009). Biogenicvolatile organic compounds in the Earth system. New Phytol.

[CR46] Loughrin JH, Potter DA, Hamilton-Kemp TR (1995). Volatile compounds induced by herbivory act as aggregation kairomones for the Japanese beetle (*Popillia japonica* Newman). J Chem Ecol.

[CR47] Low PA, McArthur C, Fisher K, Hochuli DF (2014). Elevated volatile concentrations in high-nutrient plants: do insect herbivores pay a high price for good food?. Ecol Entomol.

[CR48] Maffei ME, Gertsch J, Appendino G (2011). Plant volatiles: production, function and pharmacology. Nat Prod Rep.

[CR49] Martin JP, Beyerlein A, Dacks AM, Reisenman CE, Riffell JA, Lei H, Hildebrand JG (2011). The neurobiology of insect olfaction: sensory processing in a comparative context. Prog Neurobiol.

[CR50] McCormick AC, Unsicker SB, Gershenzon J (2012). The specificity of herbivore-induced plant volatiles in attracting herbivore enemies. Trends Plant Sci.

[CR51] McCormick AC, Boeckler AG, Köllner TG, Gershenzon J, Unsicker SB (2014). The timing of herbivore-induced volatile emission in black poplar (*Populus nigra*) and the influence of herbivore age and identity affect the value of individual volatiles as cues for herbivore enemies. BMC Plant Biol.

[CR52] McCormick AC, Irmisch S, Reiecke A, Boeckler AG, Veit D, Reichelt M, Köllner TG, Hansson BS, Gershenzon J, Unsicker SB (2014). Herbivore-induced volatile emission in black poplar: regulation and role in attracting herbivore enemies. Plant Cell Environ.

[CR53] Mumm R, Dicke M (2010). Variation in natural plant products and the attraction of bodyguards involved in indirect plant defense. Can J Zool.

[CR54] Oluwafemi S, Dewhirst SY, Veyrat N, Powers S, Bruce TJ, Caulfield JC, Pickett JA, Birkett MA (2013). Priming of production in maize of volatile organic defence compounds by the natural plant activator *cis* jasmone. PLoS One.

[CR55] Peterson SC (1988). Chemical trail marking and following by caterpillars of *Malacosoma neustria*. J Chem Ecol.

[CR56] Pichersky E, Noel JP, Dudareva N (2006). Biosynthesis of plant volatiles: nature’s diversity and ingenuity. Science.

[CR57] Ranganathan Y, Borges RM (2010). Reducing the babel in plant volatile communication: using the forest to see the trees. Plant Biol.

[CR58] Reader T, Hochuli DF (2003). Understanding gregariousness in a larval lepidopteran: the roles of host plant, predation, and microclimate. Ecol Entomol.

[CR59] Robert CA, Erb M, Duployer M, Zwahlen C, Doyen GR, Turlings TC (2012). Herbivore-induced plant volatiles mediate host selection by a root herbivore. New Phytol.

[CR60] Robert CA, Veyrat N, Glauser G, Marti G, Doyen GR, Villard N, Gaillard MD, Köllner TG, Giron D, Body M, Babst BA (2012). A specialist root herbivore exploits defensive metabolites to locate nutritious tissues. Ecol Lett.

[CR61] Roff DA (1990). The evolution of flightlessness in insects. Ecol Monogr.

[CR62] Rostás M, Hilker M (2002). Feeding damage by larvae of the mustard leaf beetle deters conspecific females from oviposition and feeding. Entomol Exp Appl.

[CR63] Scanlon JT, Willis DE (1985). Calculation of flame ionization de- tector relative response factors using the effective carbon number concept. J Chromatogr Sci.

[CR64] Schnitzler JP, Louis S, Behnke K, Loivamaki M (2010). Poplar volatiles – biosynthesis, regulation and (eco)physiology of isoprene and stress-induced isoprenoids. Plant Biol.

[CR65] Singer MS, Carriere Y, Theuring C, Hartmann T (2004). Disentangling food quality from resistance against parasitoids: diet choice by a generalist caterpillar. Am Nat.

[CR66] Tamiru A, Bruce TJ, Woodcock CM, Caulfield JC, Midega CA, Ogol CK, Mayon P, Birkett MA, Pickett JA, Khan ZR (2011). Maize landraces recruit egg and larval parasitoids in response to egg deposition by a herbivore. Ecol Lett.

[CR67] Todd JL, Baker TC, Hansson BS (1999). Funtion of peripheral olfactory organs. Insect olfaction.

[CR68] Turlings TCJ, Loughrin JH, McCall PJ, Rose USR, Lewis WJ, Tumlinson JH (1995). How caterpillar-damaged plants protect themselves by attracting parasitic wasps. Proc Natl Acad Sci U S A.

[CR69] ul Hassan MN, Zainal Z, Ismail I (2015). Green leaf volatiles: biosynthesis, biological functions and their applications in biotechnology. Plant Biotechnol J.

[CR70] von Mérey GE, Veyrat N, D’Alessandro M, Turlings TCJ (2013). Herbivore-induced maize leaf volatiles affect attraction and feeding behavior of *Spodoptera littoralis* caterpillars. Front Plant Sci.

[CR71] Webster B, Bruce T, Pickett J, Hardie J (2010). Volatiles functioning as host cues in a blend become nonhost cues when presented alone to the black bean aphid. Anim Behav.

[CR72] Yoneya K, Ozawa R, Takabayashi J (2010). Specialist leaf beetle larvae use volatiles from willow leaves infested by conspecifics for reaggregation in a tree. J Chem Ecol.

[CR73] Zalucki MP, Clarke AR, Malcolm SB (2002). Ecology and behavior of first instar larval Lepidoptera. Annu Rev Entomol.

