# Supplementary material for: Feeding Experience Affects the Behavioral Response of Polyphagous Gypsy Moth Caterpillars to Herbivore-induced Poplar Volatiles
Source: J Chem Ecol. 2016 May 12;42:382–93. doi: 10.1007/s10886-016-0698-7 (PMC4912982; doi:10.1007/s10886-016-0698-7)
Supplement: Supplementary file 1 — Detail of the setups used for behavioral assays. A. Y tube olfactometer with glass vessels, and B. Four-arm olfactometer. (DOCX 4954 kb) [file 10886_2016_698_MOESM1_ESM.docx]

A.


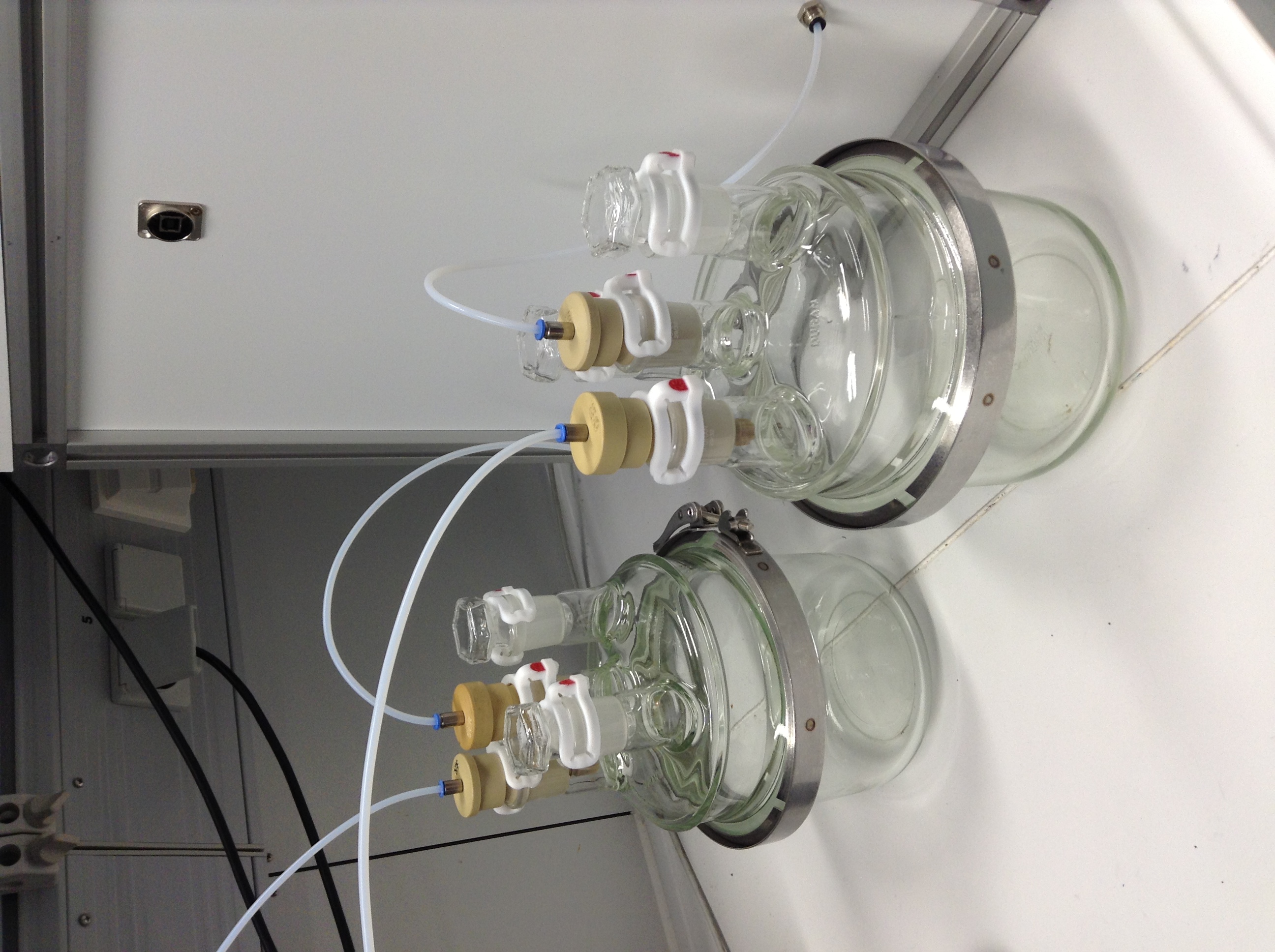

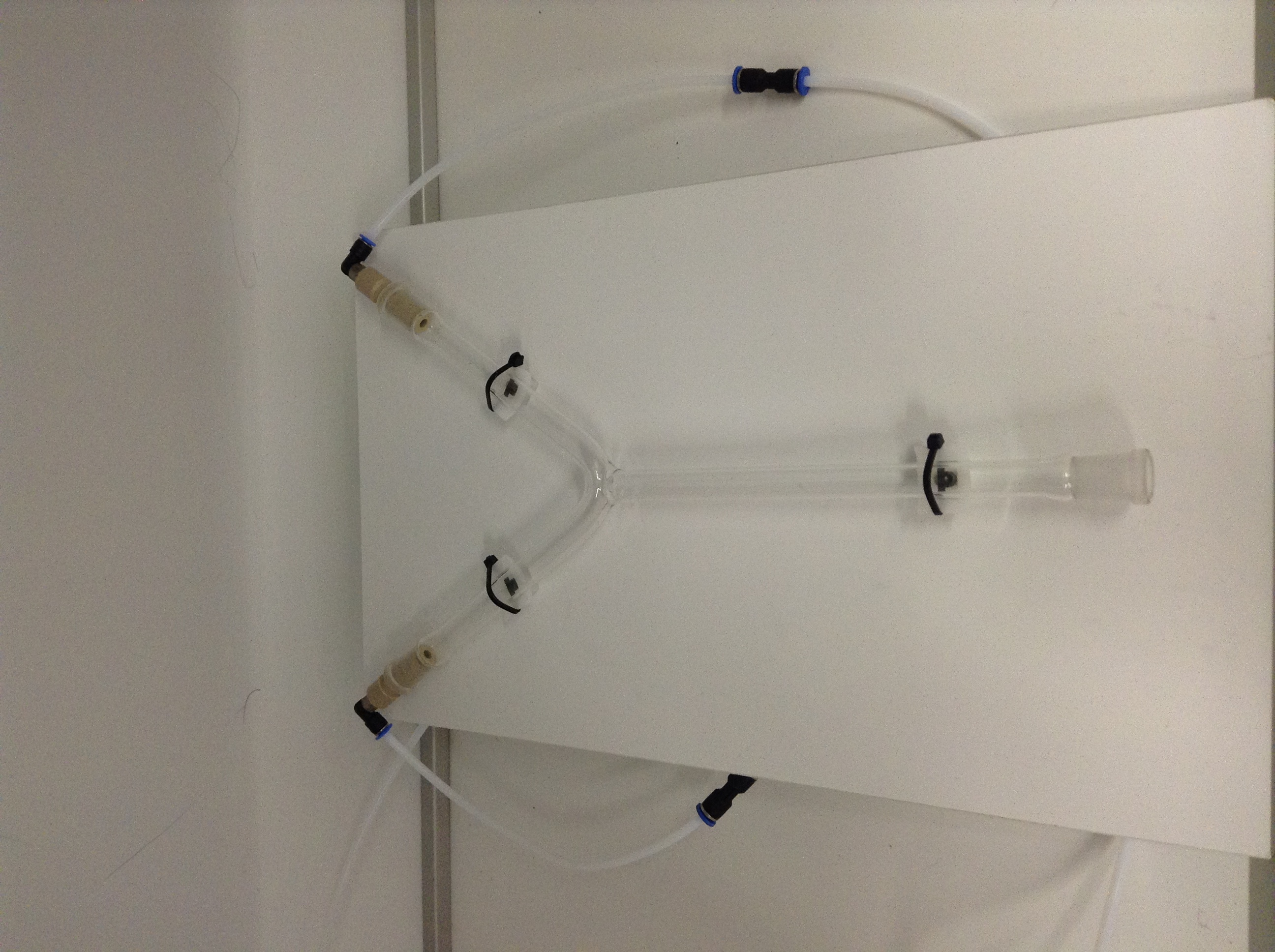


B.


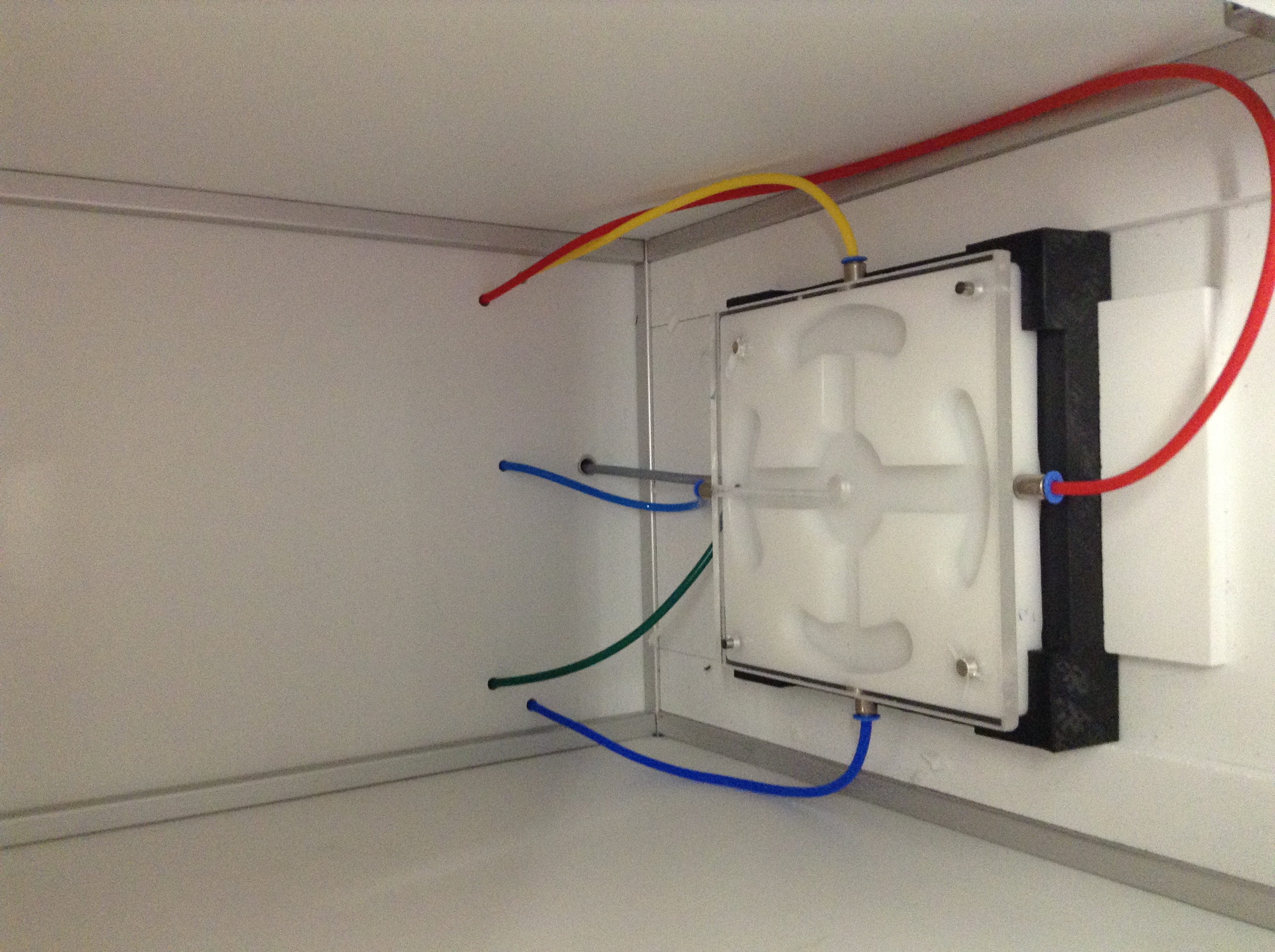


**Figure S1.**  Detail of the setups used for behavioral assays. A. Y tube olfactometer with glass vessels, and B. Four-arm olfactometer.
