# Supplementary material for: Feeding Experience Affects the Behavioral Response of Polyphagous Gypsy Moth Caterpillars to Herbivore-induced Poplar Volatiles
Source: J Chem Ecol. 2016 May 12;42:382–93. doi: 10.1007/s10886-016-0698-7 (PMC4912982; doi:10.1007/s10886-016-0698-7)
Supplement: Supplementary file 2 — Results of Chi square test for Y-tube olfactometer essays and Pearson Chi square test for comparison of behavioral responses between naïve and experienced caterpillars followed by 2 × 2 Phi-Cramer’s V test. (DOCX 15 kb) [file 10886_2016_698_MOESM2_ESM.docx]

**Table S1.** Results of *Chi square* test for Y-tube olfactometer essays and Pearson *Chi square* test for comparison of behavioral responses between naïve and experienced caterpillars followed by 2x2 *Phi-Cramer’s V* test.

|  |  | Odor preference in Y tube olfactometer essays | | | | Comparison naïve vs. experienced | | |
| --- | --- | --- | --- | --- | --- | --- | --- | --- |
| Odor pairs | Level of experience | *N* | *X^2^* | *P* | Preferred odor | *X^2^* | *P* | *Phi-Cramer’s V* |
| Undamaged plant vs. Clean air | Naïve | 43 | 3.93 | 0.047 | Undamaged plant | 0.75 | 0.385 | ± 0.091 |
|  | Experienced | 49 | 10.79 | 0.001 | Undamaged pant |  |  |  |
| Undamaged plant vs. up to 6 h damage | Naïve | 46 | 4.26 | 0.039 | 6 h damage | 0.32 | 0.569 | ± 0.062 |
|  | Experienced | 38 | 6.74 | 0.009 | 6h damage |  |  |  |
| Undamaged plant vs. 24-30 h damage | Naïve | 44 | 26.27 | <0.001 | 30 h damage | 27.73 | 0.000 | ±0.600 |
|  | Experienced | 33 | 5.12 | 0.024 | Undamaged plant |  |  |  |
| Undamaged plant vs. 24-30 h after herbivore removal | Naïve | 36 | 1.00 | 0.317 | No preference | 2.47 | 0.116 | ±0.189 |
|  | Experienced | 33 | 1.48 | 0.220 | No preference |  |  |  |
| Clean air vs. larvae and frass | Naïve | 30 | 8.53 | 0.003 | Larvae and frass | 0.34 | 0.561 | ±0.080 |
|  | Experienced | 23 | 3.52 | 0.060 | No preference |  |  |  |
