# Supplementary material for: Feeding Experience Affects the Behavioral Response of Polyphagous Gypsy Moth Caterpillars to Herbivore-induced Poplar Volatiles
Source: J Chem Ecol. 2016 May 12;42:382–93. doi: 10.1007/s10886-016-0698-7 (PMC4912982; doi:10.1007/s10886-016-0698-7)
Supplement: Supplementary file 3 — Volatile emission from leaves of undamaged poplar saplings (control) and saplings fed upon by second instar Lymantria dispar larvae (LD2) up to 6 hr (Short-term herbivory), and between 24 and 30 hr (longer-term herbivory). Values are displayed in ng g-1 FW h-1. The reason why two controls are shown is because the same plants were measured at an initial stage of herbivory (up to 6 hr of damage) and between 24 and 30 hr of damage, and so were the corresponding controls (undamaged plants). Bold compounds were used for behavioral assays. (DOCX 31 kb) [file 10886_2016_698_MOESM3_ESM.docx]

**Table S2.** Volatile emission from leaves of undamaged poplar saplings (control) and saplings fed upon by second instar *Lymantria dispar* larvae (LD2) up to 6 h (Short-term herbivory), and between 24 and 30 h (longer-term herbivory). Values are displayed in ng g^-1^ FW h^-1^. The reason why two controls are shown is because the same plants were measured at an initial stage of herbivory (up to 6 h of damage) and between 24 and 30 h of damage, and so were the corresponding controls (undamaged plants). Bold compounds were used for behavioral assays.

|  |  |  | Control (short-term) | | Short-term herbivory | | Control (longer-term) | | Longer-term herbivory | |
| --- | --- | --- | --- | --- | --- | --- | --- | --- | --- | --- |
| ID | RT | Name | MEAN | SEM | MEAN | SEM | MEAN | SEM | MEAN | SEM |
| 1 | 8.72 | α-Pinene | 12.29 | 1.08 | 10.38 | 0.41 | 7.94 | 1.25 | 7.82 | 0.60 |
| 2 | 9.62 | Camphene | 18.53 | 1.64 | 15.08 | 0.63 | 13.51 | 2.16 | 11.40 | 1.01 |
| 3 | 10.54 | β-Pinene | 12.57 | 1.12 | 9.91 | 0.47 | 8.86 | 1.46 | 7.04 | 0.65 |
| 4 | 10.85 | Sabinene | 2.66 | 0.19 | 2.24 | 0.11 | 1.75 | 0.21 | 2.86 | 0.19 |
| 5 | 11.875 | Myrcene | 4.60 | 0.40 | 3.20 | 0.23 | 3.16 | 0.32 | 3.65 | 0.34 |
| 6 | 12.79 | Limonene | 2.66 | 0.16 | 2.58 | 0.11 | 1.35 | 0.10 | 3.76 | 0.28 |
| 7 | 13.12 | Eucalyptol | 0.96 | 0.23 | 0.78 | 0.04 | 0.29 | 0.13 | 0.55 | 0.13 |
| 8 | 13.37 | 4-Penten-1-yl-acetate | 0.94 | 0.12 | 1.28 | 0.05 | 0.71 | 0.18 | 0.81 | 0.04 |
| 9 | 13.71 | (*Z*)-β-Ocimene | 10.29 | 0.81 | 8.73 | 0.26 | 8.15 | 0.89 | 12.80 | 1.10 |
| **10** | **13.98** | **Salicyl aldehyde** | **0.22** | **0.06** | **0.19** | **0.05** | **0.23** | **0.07** | **0.58** | **0.02** |
| **11** | **14.15** | **(*E*)-β-Ocimene** | **30.69** | **3.46** | **29.88** | **2.18** | **27.28** | **1.75** | **101.38** | **14.17** |
| 12 | 14.72 | Hexyl acetate | 0.97 | 0.10 | 3.00 | 0.22 | 0.45 | 0.03 | 1.91 | 0.16 |
| **13** | **15.59** | **(*E*)-DMNT** | **18.10** | **1.80** | **23.76** | **1.07** | **12.05** | **1.19** | **55.29** | **6.00** |
| **14** | **15.93** | **(*Z*)-3-Hexenyl acetate** | **101.40** | **10.94** | **335.24** | **17.84** | **49.14** | **2.65** | **137.81** | **6.01** |
| 15 | 16.31 | (*E*)-2-Hexenyl acetate | 1.14 | 0.13 | 4.89 | 0.46 | 1.81 | 0.18 | 1.27 | 0.12 |
| 16 | 16.49 | Anisole | 5.42 | 0.66 | 6.08 | 0.78 | 0.76 | 0.15 | 0.21 | 0.06 |
| 17 | 16.81 | 1-Hexanol | 1.08 | 0.17 | 0.75 | 0.05 | 0.28 | 0.08 | 0.45 | 0.09 |
| 18 | 17.35 | Alloocimene | 0.80 | 0.06 | 0.73 | 0.04 | 0.41 | 0.08 | 1.10 | 0.11 |
| **19** | **17.62** | **(*Z*)-3-Hexenol** | **10.85** | **1.78** | **25.87** | **1.13** | **4.51** | **0.52** | **17.99** | **1.57** |
| 20 | 17.83 | (Z)-3-Hexenyl butyrate | 2.42 | 0.22 | 1.74 | 0.08 | 1.26 | 0.08 | 0.76 | 0.10 |
| 21 | 17.93 | Nonanal | 0.99 | 0.19 | 2.36 | 0.07 | 1.72 | 0.08 | 2.58 | 0.19 |
| 22 | 18.17 | (*E*)-2-Hexenol | 0.40 | 0.08 | 0.98 | 0.09 | 0.13 | 0.03 | 0.69 | 0.16 |

**Table S2.** Continued

|  |  |  | Control (short-term) | | Short-term herbivory | | Control (longer-term) | | Longer-term herbivory | |
| --- | --- | --- | --- | --- | --- | --- | --- | --- | --- | --- |
| ID | RT | Name | MEAN | SEM | MEAN | SEM | MEAN | SEM | MEAN | SEM |
| 23 | 19.68 | α-cubebene | 2.44 | 0.24 | 1.60 | 0.10 | 1.01 | 0.13 | 0.96 | 0.16 |
| 24 | 20.04 | (Z)-3-Hexenyl-2-methylbutanoate | 11.95 | 1.01 | 6.84 | 0.43 | 4.79 | 0.36 | 2.53 | 0.24 |
| 25 | 20.15 | (Z)-Epoxy-ocimene | 0.51 | 0.10 | 0.86 | 0.02 | 0.08 | 0.04 | 0.61 | 0.08 |
| **26** | **20.03** | **(*Z*)-2-Methylbutyraldoxime** | **0.00** | **0.00** | **0.58** | **0.02** | **0.00** | **0.00** | **3.23** | **0.36** |
| 27 | 20.39 | (*E*)-3-Methylbutyraldoxime | 1.69 | 0.11 | 1.59 | 0.06 | 0.41 | 0.08 | 1.02 | 0.07 |
| 28 | 20.85 | (*E*)-2-Methylbutyraldoxime | 0.00 | 0.00 | 0.12 | 0.05 | 0.00 | 0.00 | 0.81 | 0.12 |
| 29 | 21.15 | Camphor | 5.06 | 0.32 | 5.38 | 0.29 | 4.13 | 0.76 | 3.35 | 0.25 |
| 30 | 21.15 | Benzaldehyde | 0.58 | 0.09 | 0.39 | 0.07 | 1.29 | 0.07 | 1.06 | 0.13 |
| 31 | 21.47 | (*Z*)-3-Methylbutyraldoxime | 1.12 | 0.08 | 1.01 | 0.04 | 0.48 | 0.13 | 1.40 | 0.18 |
| **32** | **21.75** | **Linalool** | **0.98** | **0.09** | **1.24** | **0.17** | **1.33** | **0.20** | **3.56** | **0.39** |
| *33* | 22.61 | Nonyl acetate (internal standard) | IS | IS | IS | IS | IS | IS | IS | IS |
| 34 | 22.98 | β -Elemene | 1.79 | 0.11 | 1.95 | 0.16 | 1.75 | 0.16 | 1.20 | 0.06 |
| **35** | **23.22** | **(*E*)-β-Caryophyllene** | **14.08** | **0.17** | **18.08** | **0.33** | **16.91** | **0.56** | **24.54** | **0.81** |
| 36 | 24.03 | β -Cubebene | 0.79 | 0.10 | 1.27 | 0.09 | 0.25 | 0.07 | 0.70 | 0.27 |
| 37 | 24.18 | α-Cubebene | 0.68 | 0.08 | 0.42 | 0.08 | 0.27 | 0.07 | 0.00 | 0.00 |
| 38 | 24.37 | Alloaromadendrene | 1.57 | 0.09 | 1.98 | 0.15 | 1.46 | 0.10 | 0.44 | 0.08 |
| 39 | 24.5 | unidentified | 0.85 | 0.09 | 1.78 | 0.15 | 0.26 | 0.07 | 0.00 | 0.00 |
| 40 | 24.67 | (*E*)-β-Farnesene | 0.00 | 0.00 | 0.63 | 0.12 | 0.00 | 0.00 | 0.00 | 0.00 |
| 41 | 24.9 | α-Humulene | 23.19 | 1.29 | 24.40 | 1.93 | 17.93 | 1.63 | 10.39 | 0.67 |
| 42 | 25.51 | Borneol | 2.47 | 0.20 | 2.97 | 0.17 | 2.79 | 0.42 | 1.51 | 0.13 |

**Table S2.** Continued

|  |  |  | Control (short-term) | | Short-term herbivory | | Control (longer-term) | | Longer-term herbivory | |
| --- | --- | --- | --- | --- | --- | --- | --- | --- | --- | --- |
| ID | RT | Name | MEAN | SEM | MEAN | SEM | MEAN | SEM | MEAN | SEM |
| 43 | 25.8 | Germacrene D | 9.24 | 0.72 | 9.60 | 0.48 | 7.50 | 0.65 | 17.55 | 1.85 |
| 44 | 26.04 | (*E*)-α-Bergamotene | 1.17 | 0.10 | 1.34 | 0.07 | 0.85 | 0.12 | 0.14 | 0.06 |
| 45 | 26.09 | α-Muurolene | 2.39 | 0.16 | 2.33 | 0.11 | 2.55 | 0.23 | 1.56 | 0.13 |
| 46 | 26.55 | (*E,E*)- α-Farnesene | 3.29 | 0.71 | 1.63 | 0.10 | 2.12 | 0.25 | 10.98 | 2.30 |
| 47 | 26.82 | δ-Cadinene | 5.35 | 0.30 | 5.59 | 0.37 | 7.68 | 0.82 | 3.95 | 0.26 |
| 48 | 26.89 | γ-Cadinene | 2.53 | 0.12 | 2.68 | 0.15 | 3.34 | 0.34 | 1.55 | 0.09 |
| 49 | 26.98 | Methyl salicylate | 0.18 | 0.05 | 0.25 | 0.07 | 0.25 | 0.07 | 0.10 | 0.04 |
| 50 | 27.61 | α-Cadinene | 0.49 | 0.05 | 0.28 | 0.08 | 0.48 | 0.10 | 0.09 | 0.04 |
| 51 | 27.86 | (*E,E*)-TMTT | 2.53 | 0.28 | 2.30 | 0.18 | 3.20 | 0.28 | 4.48 | 0.56 |
| 52 | 27.96 | unidentified | 1.14 | 0.05 | 1.29 | 0.05 | 1.15 | 0.08 | 0.77 | 0.10 |
| 53 | 29.21 | Benzyl alcohol | 0.43 | 0.09 | 0.49 | 0.06 | 0.94 | 0.22 | 1.26 | 0.22 |
| 54 | 29.97 | Benzene ethanol | 0.53 | 0.08 | 0.47 | 0.09 | 1.31 | 0.09 | 1.63 | 0.31 |
| **55** | **30.27** | **Benzyl cyanide** | **0.26** | **0.05** | **1.08** | **0.06** | **0.41** | **0.08** | **8.82** | **1.32** |
| 56 | 30.77 | (*Z*)-Jasmone | 0.53 | 0.08 | 0.41 | 0.09 | 0.48 | 0.09 | 0.71 | 0.10 |
| 57 | 31.69 | Caryophyllene oxide | 0.83 | 0.06 | 0.79 | 0.10 | 1.25 | 0.17 | 0.41 | 0.12 |
| 58 | 32.32 | γ-Terpinene | 1.14 | 0.18 | 2.11 | 0.17 | 1.45 | 0.14 | 1.27 | 0.09 |
| 59 | 32.32 | unidentified | 0.27 | 0.12 | 0.00 | 0.00 | 0.66 | 0.06 | 1.59 | 0.17 |
| 60 | 32.79 | Humulene oxide | 1.03 | 0.06 | 0.87 | 0.11 | 1.60 | 0.12 | 0.86 | 0.13 |
| 61 | 33.05 | Nerolidol | 2.38 | 0.22 | 3.04 | 0.31 | 5.98 | 0.69 | 3.08 | 0.36 |
| 62 | 34.06 | Phenylnitroethane | 0.42 | 0.10 | 0.00 | 0.00 | 0.54 | 0.10 | 1.88 | 0.28 |
| **63** | **34.95** | **Eugenol** | **0.23** | **0.06** | **1.03** | **0.09** | **1.74** | **0.12** | **3.89** | **0.48** |
| 64 | 35.18 | τ-muurolool | 0.49 | 0.07 | 0.21 | 0.06 | 1.53 | 0.14 | 0.69 | 0.09 |
