# Supplementary material for: Feeding Experience Affects the Behavioral Response of Polyphagous Gypsy Moth Caterpillars to Herbivore-induced Poplar Volatiles
Source: J Chem Ecol. 2016 May 12;42:382–93. doi: 10.1007/s10886-016-0698-7 (PMC4912982; doi:10.1007/s10886-016-0698-7)
Supplement: Supplementary file 4 — Results of a Wilcoxon signed rank test on the time spent by gypsy moth larvae in areas of an olfactometer permeated with a solvent or the tested odor and U Mann–Whitney test for comparison between naïve and experienced caterpillar behavior. Z and P values are given for comparisons between the odor and control treated arms for each level of experience (naïve or experienced) tested independently, and U and P values are given for comparisons between experienced and naïve caterpillars for each compound. (DOCX 17 kb) [file 10886_2016_698_MOESM4_ESM.docx]

**Table S3.** Results of a Wilcoxon signed rank test on the time spent by gypsy moth larvae in areas of an olfactometer permeated with a solvent or the tested odor and *U* Mann-Whitney test for comparison between naïve and experienced caterpillar behavior. *Z* and *P* values are given for comparisons between the odor and control treated arms for each level of experience (naïve or experienced) tested independently, and *U* and *P* values are given for comparisons between experienced and naïve caterpillars for each compound.

| N | Compound | Level of  experience | *Z*  (Wilcoxon) | *P*  (two tailed) | *U* (Mann-Whitney) | *P*  (two tailed) |
| --- | --- | --- | --- | --- | --- | --- |
| 24 | 2-Methylbutyraldoxime | Naïve | -0.514 | 0.607 | 262 | 0.763 |
| 23 |  | Experienced | -0.213 | 0.831 |  |  |
| 21 | Benzyl cyanide | Naïve | -2.138 | 0.033 | 227.5 | 0.932 |
| 22 |  | Experienced | -1.997 | 0.046 |  |  |
| 24 | (*E*)-β-Caryophyllene | Naïve | -0.771 | 0.440 | 281.5 | 0.708 |
| 25 |  | Experienced | -0.256 | 0.798 |  |  |
| 24 | DMNT | Naïve | -0.629 | 0.530 | 186 | 0.022 |
| 25 |  | Experienced | -2.623 | 0.009 |  |  |
| 25 | (*E*)-β-Ocimene | Naïve | -1.466 | 0.143 | 129.2699 | 0.861 |
| 25 |  | Experienced | -0.417 | 0.677 |  |  |
| 24 | Eugenol | Naïve | -0.114 | 0.909 | 261.5 | 0.584 |
| 24 |  | Experienced | -0.057 | 0.954 |  |  |
| 21 | Linalool | Naïve | -0.643 | 0.520 | 255.5 | 0.877 |
| 25 |  | Experienced | -0.955 | 0.399 |  |  |
| 24 | Salicyl aldehyde | Naïve | -2.029 | 0.043 | 264 | 1 |
| 22 |  | Experienced | -1.834 | 0.067 |  |  |
| 24 | (*Z*)-3-Hexenol | Naïve | -2.257 | 0.024 | 259 | 0.717 |
| 23 |  | Experienced | -0.973 | 0.330 |  |  |
| 22 | (*Z*)-3-Hexenyl acetate | Naïve | -2.159 | 0.031 | 237 | 0.907 |
| 22 |  | Experienced | -2.256 | 0.024 |  |  |
